# Dopamine Neurons That Cotransmit Glutamate, From Synapses to Circuits to Behavior

**DOI:** 10.3389/fncir.2021.665386

**Published:** 2021-05-19

**Authors:** Daniel Eskenazi, Lauren Malave, Susana Mingote, Leora Yetnikoff, Samira Ztaou, Vlad Velicu, Stephen Rayport, Nao Chuhma

**Affiliations:** ^1^Department of Psychiatry, Columbia University, New York, NY, United States; ^2^Department of Molecular Therapeutics, New York State Psychiatric Institute, New York, NY, United States; ^3^Neuroscience Initiative, Advanced Science Research Center, Graduate Center of The City University of New York, New York, NY, United States; ^4^Department of Psychology, College of Staten Island, City University of New York, Staten Island, NY, United States; ^5^CUNY Neuroscience Collaborative, The Graduate Center, City University of New York, New York, NY, United States

**Keywords:** VGLUT2, VMAT2, glutaminase, schizophrenia, addiction, psychostimulant, Parkinson disease

## Abstract

Discovered just over 20 years ago, dopamine neurons have the ability to cotransmit both dopamine and glutamate. Yet, the functional roles of dopamine neuron glutamate cotransmission and their implications for therapeutic use are just emerging. This review article encompasses the current body of evidence investigating the functions of dopamine neurons of the ventral midbrain that cotransmit glutamate. Since its discovery in dopamine neuron cultures, further work *in vivo* confirmed dopamine neuron glutamate cotransmission across species. From there, growing interest has led to research related to neural functioning including roles in synaptic signaling, development, and behavior. Functional connectome mapping reveals robust connections in multiple forebrain regions to various cell types, most notably to cholinergic interneurons in both the medial shell of the nucleus accumbens and the lateral dorsal striatum. Glutamate markers in dopamine neurons reach peak levels during embryonic development and increase in response to various toxins, suggesting dopamine neuron glutamate cotransmission may serve neuroprotective roles. Findings from behavioral analyses reveal prominent roles for dopamine neuron glutamate cotransmission in responses to psychostimulants, in positive valence and cognitive systems and for subtle roles in negative valence systems. Insight into dopamine neuron glutamate cotransmission informs the pathophysiology of neuropsychiatric disorders such as addiction, schizophrenia and Parkinson Disease, with therapeutic implications.

## Introduction

Dopamine (DA) neurons were first identified by their monoamine content, and then by the expression of the DA synthetic enzyme tyrosine hydroxylase (TH) (for review see Iversen and Iversen, 2007). Heterogeneity of DA neurons was first recognized as mediolateral differences between ventral tegmental area (VTA) and substantia nigra (SN) DA neurons (for reviews on this topic see Grace et al., 2007; Liss and Roeper, 2008). DA neurons, like most central nervous system neurons, use multiple neurotransmitters (Kupfermann, 1991), adding a further dimension of heterogeneity. Peptide cotransmission was recognized first, with evidence that DA neurons use cholecystokinin and neurotensin as cotransmitters (Hökfelt et al., 1980; Gonzalez-Reyes et al., 2012).

**FIGURE 1:**
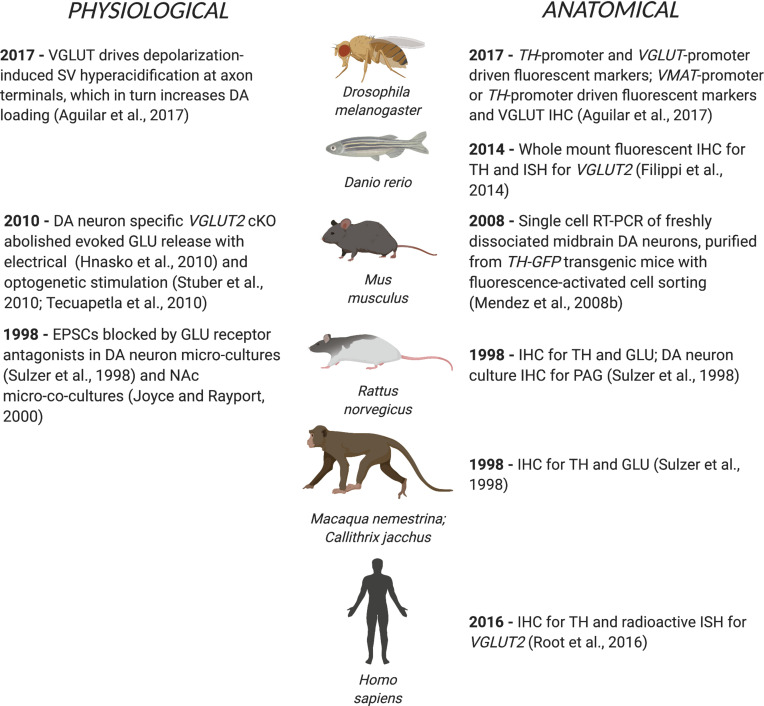
DA neuron GLU cotransmission spans phylogeny from flies to humans. The first physiological or anatomical evidence for DA neuron GLU cotransmission is cited by species.

Cotransmission involving two small molecule neurotransmitters — especially with competing synaptic actions — was recognized more recently (for review see Hnasko and Edwards, 2012). DA neuron glutamate (GLU) cotransmission was first shown in single-cell microcultures of identified rat DA neurons (Sulzer et al., 1998). Electrical stimulation of genetically tagged DA neurons in quasi-horizontal mouse brain slices revealed DA neuron GLU cotransmission in the ventral striatum (Chuhma et al., 2004) and its frequency dependent modulation by concomitantly released DA (Chuhma et al., 2009). Optogenetic stimulation of DA neuron terminals showed that DA neurons make monosynaptic GLU connections to spiny projection neurons (SPNs) in the nucleus accumbens (NAc) (Stuber et al., 2010; Tecuapetla et al., 2010). DA neurons cotransmitting GLU (DA-GLU neurons) require both vesicular monoamine transporter 2 (VMAT2) for DA release (Fon et al., 1997) and vesicular glutamate transporter 2 (VGLUT2 for protein, *VGluT2* for gene and mRNA) for GLU release (Dal Bo et al., 2004; Hnasko et al., 2010; Stuber et al., 2010). DA neurons also use GABA as a small molecule cotransmitter (for reviews see Tritsch et al., 2012; Granger et al., 2017). DA neuron GLU cotransmission extends from fruit flies to humans (Figure 1), arguing for important physiological roles.

This review focuses on DA neuron GLU cotransmission and addresses the key questions: (1) Where do DA-GLU neurons project? (2) Are DA and GLU released together or separately? (3) What are the synaptic functions of DA neuron GLU cotransmission? (4) What are the developmental roles of DA neuron GLU cotransmission? (5) How are DA-GLU neurons affected by DA neuron toxins? (6) What are the behavioral roles of DA neuron GLU cotransmission? (7) Does DA neuron GLU cotransmission have a role in human disorders?

## Where Do DA-GLU Neurons Project?

### DA-GLU Neurons in the Ventral Midbrain

Dopamine neurons in the ventral midbrain are divided between the VTA and SN. DA-GLU neurons show a medial preponderance, are mainly in the VTA, and project predominantly to the ventral striatum/NAc (Li et al., 2013; Morales and Root, 2014; Yamaguchi et al., 2015; Zhang et al., 2015; Root et al., 2016; Chuhma et al., 2018; Poulin et al., 2018; Mingote et al., 2019). DA-GLU neurons are identified by *TH* and *VGluT2* expression. Expression of *VGluT2* in DA neurons is necessary and sufficient to enable GLU cotransmission (Takamori et al., 2000). Indeed, DA-neuron-specific *VGluT2* cKO eliminated GLU-cotransmission synaptic responses (Stuber et al., 2010). Visualizing *VGluT2* expression in cell bodies requires *in situ* hybridization (ISH) or ectopic reporter expression driven by the *VGluT2* promoter, as VGLUT2 is rapidly exported to axon terminals. The number of DA-GLU (i.e., TH^+^/VGLUT2^+^) neurons varies across the lifespan, species, brain region and study (Table 1). In the VTA, DA-GLU neurons account for 10-30% of DA neurons, and are most abundant in the interfascicular nucleus (IF), the central linear nucleus (CLi), the rostral linear nucleus (RLi), and the parabrachial pigmented nucleus (PBP) (Kawano et al., 2006; Li et al., 2013). In the SN, DA-GLU neurons account for about 5–10%, and are most abundant in the dorsal SN pars compacta (SNc) and the pars lateralis in rodents, as well as primates including humans (Yamaguchi et al., 2013; Root et al., 2016; Steinkellner et al., 2018).

**TABLE 1:** *TH* and *VGluT2* coexpression in midbrain DA neurons.

				TH^+^VGLUT2^+^/Total TH^+^%							
Age	Species	Genotype	Method	Midbrain (Total)	Medial-only				Lateral-only		Citation
E11	Mouse	WT	ISH		(>E14)						Dumas and Wallén-Mackenzie, 2019
E14	Mouse	WT	ISH		(<E11)						
E14	Mouse	*TH*^*EGFP*^	sc RT-PCR	7							Fortin et al., 2012
E16				47							
E18				33							
E15, 16	Rat	WT	ISH	(High)							Dal Bo et al., 2008
E18, 21				(Low)							
P0	Mouse	*TH*^*EGFP*^	dissociation, sc RT-PCR	25							Mendez et al., 2008b
P0	Mouse	*VGluT2*^*EGFP*^ bacterial artificial chromosome	IHC (TH, EGFP)	2							
P0-2	Mouse	*TH-EGFP*	sc RT-PCR	22	36				13		Fortin et al., 2012
P5	Rat	WT	ISH		3				<1		Dal Bo et al., 2008
P10	Mouse	*VGluT2*^*EGFP*^ bacterial artificial chromosome	IHC (TH, EGFP)	1							Mendez et al., 2008b
P10	Rat	WT	ISH		2				<1		Dal Bo et al., 2008
P14	Mouse	*TH*^*EGFP*^	sc RT-PCR	14							Fortin et al., 2012
P14	Mouse	*TH*^*EGFP*^	sc RT-PCR		18				14		Mendez et al., 2008b
P15	Rat	WT	ISH		2				<1		Dal Bo et al., 2008
P35	Mouse	*TH*^*EGFP*^	sc RT-PCR	30							Fortin et al., 2012
P45	Mouse	*TH*^*EGFP*^	dissociation, sc RT-PCR	14							Mendez et al., 2008b
P45	Mouse	*VGluT2*^*EGFP*^ bacterial artificial chromosome	IHC (TH, EGFP)	<1							
6–24 weeks	Mouse	WT	ISH (RNA Scope)		56		37				Yan et al., 2018
					*Medial VTA*		*Lateral VTA*				
P70	Mouse	*TH*^*EGFP*^	sc RT-PCR	47	78				25		Fortin et al., 2012
P90	Rat	WT	ISH		2				<1		Bérubé-Carrière et al., 2009
8–12 weeks	Mouse	*DAT^*IRES–Cre*^; VGluT2*^*flox/+*^	ISH		15				20		Shen et al., 2018
Adult	Rat	WT	ISH		<1						Yamaguchi et al., 2007
Adult	Rat	WT	ISH		*PBP*	3	*IF*	22			Kawano et al., 2006
					*PN*	5	*CLi*	22			
					*A10*	19	*RLi*	53			
Adult	Rat	WT	TH-IHC, *VGluT2*-ISH	*Medial PBP*		60	*IF*	10			Li et al., 2013
				*medial PN*		50	*RLi*	60			
			Laser micro-dissection, sc RT-PCR		*medial PBP*			42			
							*RLi*	42			
							*IF*	57			
Adult	Marmoset	WT	TH-IHC, *VGluT2*-ISH	*PBP*		23	*IF*	2	*Ventral SNc*	<1	Root et al., 2016
				*PN*		2	*CLi*	2	*Dorsal SNc*	<1	
				*Caudal VTA*		5	*RLi*	3	*Medial SNc*	<1	
				*Rostral VTA*		6	*PIF*	4	*Lateral SNc*	< 1	
Adult (55 years)	Human	WT	TH-IHC, *VGluT2*-ISH		*PBP*	17	*VTA*	10	*Ventral SNc*	2	
					*PN*	2	*RLi*	10	*Dorsal SNc*	3	
					*VTA subdivision*			10	*Medial SNc*	3	
									*Lateral SNc*	<1	

### DA-GLU Projections

Combinatorial intersectional genetic strategies (Fenno et al., 2014, 2020) have enabled visualization of DA-GLU neurons and their projections (Poulin et al., 2018). This has confirmed that DA-GLU neurons comprise about 30% of VTA neurons (Poulin et al., 2018; Mingote et al., 2019) and send dense projections to the NAc medial shell (m-shell), discrete, dense, column-like projections to the olfactory tubercle (OT), and sparse projections to the prefrontal cortex (PFC), mostly to deeper layers of the infralimbic and prelimbic cortices (Poulin et al., 2018). Particularly in the dorsal portion of the m-shell, all TH^+^ fibers are VGLUT2^+^, indicating that DA neuron projections in this region are predominantly from DA-GLU neurons, consistent with recent retrograde tracer studies (Mongia et al., 2019). DA-GLU neurons in the lateral SNc project to the lateral dorsal striatum with denser projections to the caudal striatum, or tail (Poulin et al., 2018). SNc DA-GLU neurons also project to the central nucleus of the amygdala (CeA), the lateral part of the capsular division, and sparsely to the ventral-most lateral nucleus and the posterior nucleus, as well as to DA islands in the entorhinal cortex (EntC) (Poulin et al., 2018; Mingote et al., 2019). Thus, DA-GLU neurons have discrete, but widely distributed forebrain projections.

### Physiological Connectivity of DA-GLU Neurons

**FIGURE 2:**
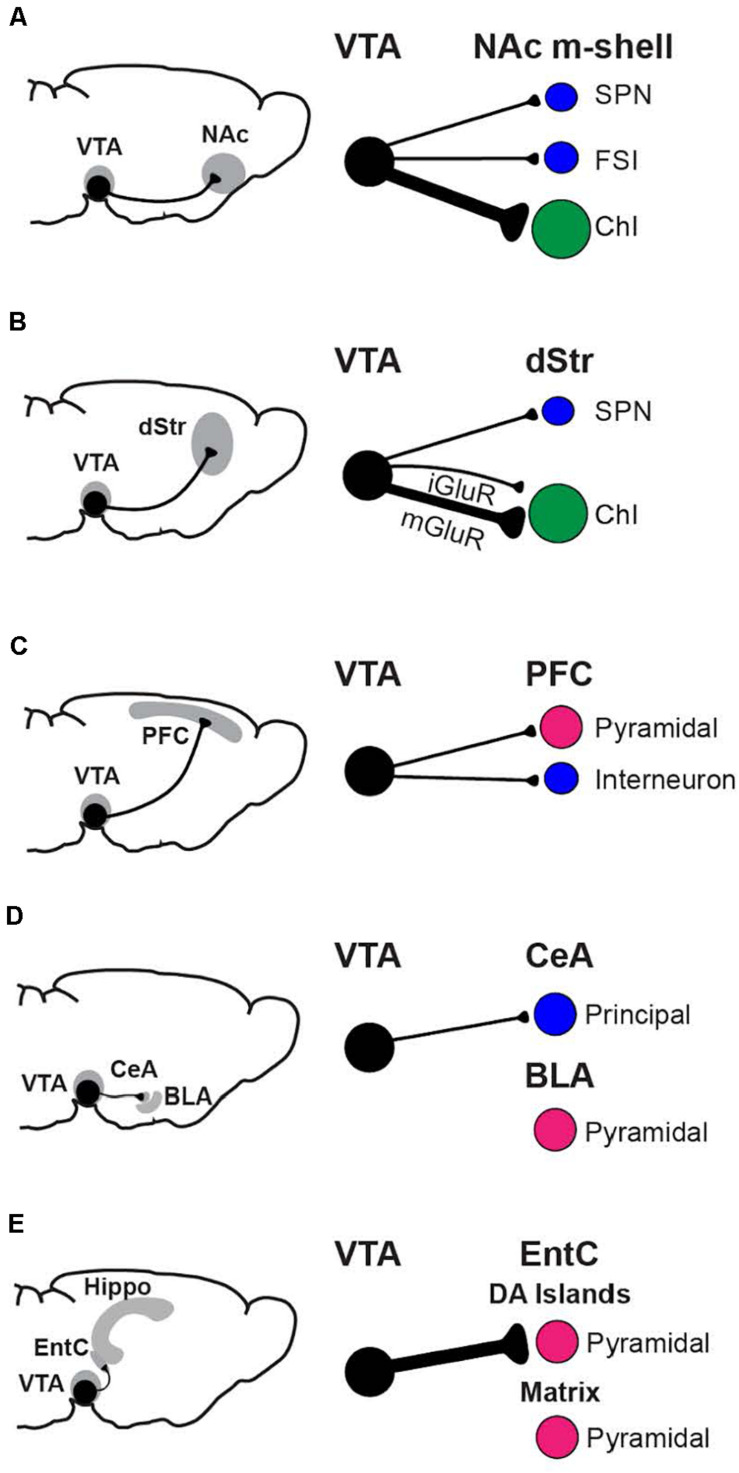
Functional connectome analysis of VTA DA neuron GLU cotransmission. Regions with prominent connections, the NAc m-shell **(A)**, dorsal striatum **(B)**, prefrontal cortex **(C)**, amygdala **(D)**, and hippocampal formation **(E)** are shown, with the neurons principally targeted by DA-GLU neurons in each region. The strength of connections is indicated by the thickness of the axons (black lines). Postsynaptic neurons are GABAergic (blue), GLUergic (magenta), or cholinergic (green).

Functional connectome mapping has addressed how the projections of DA-GLU neurons translate to their synaptic actions (Mingote et al., 2015a). *Functional connectome mapping* is the systematic recording of the strength and incidence of monosynaptic connections to identified postsynaptic neurons by optogenetic stimulation of genetically defined presynaptic neuron populations (Chuhma et al., 2011; Chuhma, 2015, 2021; Eskenazi et al., 2019). DA neurons make the most robust GLU connections in the ventral striatum, in the NAc core and shell, and the OT (Wieland et al., 2014), in accordance with the densest DA-GLU neuron projections (Poulin et al., 2018; Mingote et al., 2019; Figure 2). In the NAc m-shell, DA-GLU neurons elicit fast glutamatergic EPSCs mediated by inotropic GLU receptors (iGluR) in all SPNs, fast-spiking interneurons (FSIs) and cholinergic interneurons (ChIs), with the strongest in ChIs (Chuhma et al., 2014). In the lateral dorsal striatum, the strongest iGluR EPSCs are seen in striatonigral SPNs (Cai and Ford, 2018; Chuhma et al., 2018), and weaker EPSCs in ChIs. In addition, DA-GLU neurons elicit slower EPSCs mediated by metabotropic GLU receptors (mGluRs) in lateral dorsal striatum ChIs (Straub et al., 2014; Cai and Ford, 2018; Chuhma et al., 2018). Outside the striatum, EPSCs are seen occasionally in pyramidal neurons of layers II-III in cingulate cortex (CingC) (Mingote et al., 2015a), and in GABA interneurons in the PFC, contributing to disynaptic inhibition of pyramidal neurons (Kabanova et al., 2015; Pérez-López et al., 2018). DA-GLU neurons projecting to the cortex are mainly located in the RLi, PBP, and rostral VTA (Gorelova et al., 2012). In the EntC, DA-GLU neurons elicit EPSCs in pyramidal neurons in DA islands, while they make no connections in the hippocampus (Mingote et al., 2015a). In line with higher levels of *VGluT2* expression in DA neurons projecting to the amygdala (Taylor et al., 2014; Poulin et al., 2018), DA-GLU neurons target the CeA but not the basolateral amygdala (BLA) (Mingote et al., 2015a). Of note, most of these studies have been performed on brain slices from juvenile mice; thus, future studies on mice in early life or late adulthood may differ since the proportion of DA neurons expressing *VGluT2* may change with age (see below). In summary, DA-GLU neurons connect to different cell types in different target regions, with the highest incidence of connectivity in the NAc m-shell and lateral dorsal striatum and the largest EPSCs in the EntC.

## Are DA and GLU Released Together or Separately?

**FIGURE 3:**
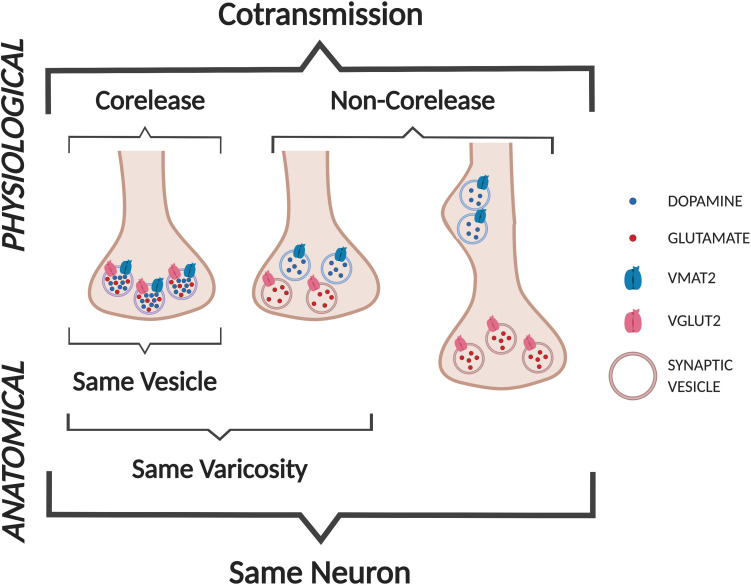
Cotransmission configurations. We define DA neuron GLU *cotransmission* as the release of DA and GLU from the same neuron. Anatomically, DA and GLU could be released from the same vesicles (labeled as *corelease*), or from separate sites in the same varicosity, or more distant sites within the same axon (not shown).

Cotransmission can be viewed as a physiological/functional property that may arise from several anatomical/structural arrangements (Figure 3). Here we use the definitions of *cotransmission* as the release of multiple different neurotransmitters from the same neuron, and *corelease* as the release of different neurotransmitters from the same synaptic vesicle (SV) (Vaaga et al., 2014; Svensson et al., 2018). Furthermore, SVs with different neurotransmitters may colocalize within the same varicosity, or segregate to different varicosities of the same neuron (e.g., some at symmetric synapses, others at asymmetric synapses).

**FIGURE 4:**
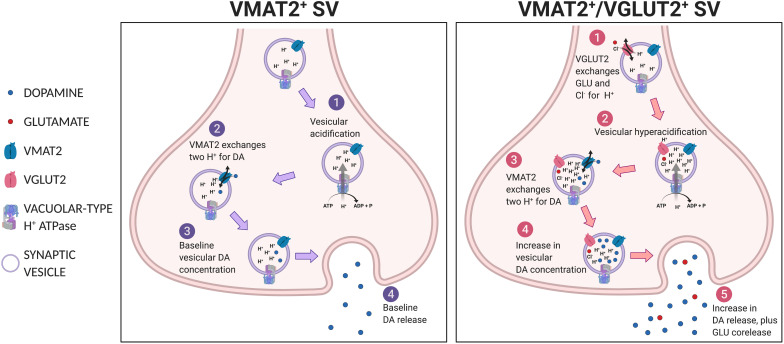
Vesicular synergy. Shown in the left panel, a VMAT2^+^ SV undergoes **(1)** vesicular acidification, then **(2)** VMAT2 exchanges two H^+^s for DA to achieve **(3)** baseline vesicular DA concentration and subsequent **(4)** baseline DA release. Shown in the right panel, a VGLUT2^+^/VMAT2^+^ co-expressing SV, **(1)** VGLUT2 transports GLU and Cl^–^ into SV, which potentiates **(2)** vacuolar-type H^+^ATPase to hyperacidify the SV, thus **(3)** more DA is drawn in via VMAT2 in exchange for protons, resulting in **(4)** greater intravesicular DA concentration and subsequent release **(5)**.

For corelease of DA and GLU, individual SVs must have both VMAT2 and VGLUT2. Co-immunoprecipitation with anti-VMAT2 and anti-VGLUT2 antibodies identified a population of striatal SVs consistent with corelease (Hnasko et al., 2010), although not in a subsequent study (Zhang et al., 2015). Uptake of GLU into a SV may potentiate the uptake and subsequent release of DA (Hnasko and Edwards, 2012; Aguilar et al., 2017), via vesicular synergy (Gras et al., 2008; Amilhon et al., 2010; El Mestikawy et al., 2011). *Vesicular synergy* refers to corelease where one neurotransmitter potentiates the uptake of another neurotransmitter in the same SV (El Mestikawy et al., 2011). VGLUT2 cotransports GLU with a single Cl^–^ into SVs in exchange for a single H^+^, thereby increasing negative charge inside SVs (Maycox et al., 1988; Cidon and Sihra, 1989) (Figure 4). This negative charge drives vacuolar-type H^+^-ATPase to increase inward flux of protons, causing SV acidification (Blakely and Edwards, 2012). In turn, DA enters SVs via VMAT2 in exchange for two H^+^ (Johnson, 1988), resulting in increased intravesicular DA concentration, and increased vesicular DA upon release. Vesicular synergy in DA neuron SVs has been shown by changes in intravesicular pH in response to both DA and GLU gradients (Hnasko et al., 2010; Aguilar et al., 2017). In mouse striatal slices, VGLUT2-dependent SV acidification is associated with increased DA release (Aguilar et al., 2017). *DAT^*Cre*^;VGluT2^*flox/flox*^* cKO mice show less striatal DA release (Stuber et al., 2010; Alsiö et al., 2011) and injections of an AAV-*Cre* viral vector into the VTA of *VGluT2*^*flox/flox*^ mice showed diminished SV acidification (Aguilar et al., 2017). These observations argue for corelease, as they require both VGLUT2 and VMAT2 in the same SV.

Anatomically, DA and GLU release sites appear to be segregated. In rats, anterograde tracing from the SN revealed two types of DA neuron synapses in the striatum (Hattori et al., 1991). Symmetric synapses were seen in TH^+^ varicosities in *en passant* configuration, consistent with sites of DA release; asymmetric synapses were located in TH^–^ axon terminals, consistent with the release of a non-DA excitatory neurotransmitter. Immunostaining of microcultures of single DA neurons showed that DA neurons have partially overlapping populations of TH^+^ and GLU^+^ varicosities (Sulzer et al., 1998). Several subsequent ultrastructural studies have found sparse TH^+^/VGLUT2^+^ varicosities in rat (Bérubé-Carrière et al., 2009; Moss et al., 2011) and mouse striata (Bérubé-Carrière et al., 2012; Fortin et al., 2019). VMAT2 and VGLUT2 appear to be actively trafficked to different processes; VMAT2 overexpression does not reduce segregation, consistent with an active process that mediates spatial segregation (Zhang et al., 2015). DA neurons co-cultured with ventral striatal neurons demonstrated enhanced segregation of TH^+^ and VGLUT2^+^ varicosities, suggesting that target-dependent factors may influence *VGluT2* expression and/or VGLUT2 localization (Fortin et al., 2019).

Although DA transients and cotransmitted GLU EPSCs elicited by optogenetic stimulation share similar release properties (Adrover et al., 2014), more recent functional studies support segregation of DA and GLU release. DA and GLU release by optogenetic stimulation deplete with different kinetics, are coupled to different types of presynaptic Ca^2+^ channels, and are differentially coupled to active zone proteins (adaptor protein 3, synaptic vesicle protein 2 and piccolo) (Silm et al., 2019). These findings are consistent with spatial segregation of DA and GLU SVs. However, studies in *Drosophila* demonstrate that a single VGLUT protein is sufficient to fill a SV with GLU (Daniels et al., 2006); thus, *VGluT2* expression levels with a physiological impact may be below the detection threshold of some methods under certain conditions, e.g., immunohistochemistry (IHC) under electron microscopy. Ultimately, while low levels of VGLUT2 in VMAT2-containing SVs may mediate corelease, spatial segregation of DA and GLU release sites appears to be the predominant configuration in DA-GLU neurons.

## What Are the Synaptic Functions of DA Neuron GLU Cotransmission?

### Excitatory Synaptic Transmission

DA volume transmission — where DA is released at non-synaptic sites and diffuses to extra-synaptic receptors — signals on a slower time frame than direct synaptic connections (Sulzer et al., 2016). In contrast, GLU cotransmission via direct synaptic connections operates on a faster time frame and conveys a discrete signal (though GLU can also act on a slower time scale at extrasynaptic sites via mGluRs). In NAc m-shell ChIs, optogenetic stimulation of DA neuron axons elicits a burst mediated by iGluRs, followed by a post-burst hyperpolarization mediated mainly by small conductance Ca^2+^-dependent K^+^ channels and partially by D2 receptors (Chuhma et al., 2014). In lateral dorsal striatum ChIs, the response is a pause mediated by D2 receptors followed by excitation mediated by mGluR1 and D1/5 receptors coupling to transient receptor potential channels 3 and 7 (Cai and Ford, 2018; Chuhma et al., 2018).

Dopamine neuron GLU EPSCs are subject to frequency-dependent DA modulation. In the NAc m-shell, DA causes counteracting D2-mediated presynaptic inhibition and D1-mediated postsynaptic facilitation through closure of K^+^ channels on GLU cotransmission. At *tonic-firing* frequencies D2-mediated presynaptic inhibition dominates and GLU responses are attenuated, while at *burst-firing* frequencies postsynaptic facilitation dominates and the GLU responses are enhanced (Chuhma et al., 2009). DA neuron GLU EPSPs are attenuated subsequent to low-dose amphetamine, whereas high-dose amphetamine attenuates fast DA transmission as well (Chuhma et al., 2014).

### Circuit-Level Effects

**FIGURE 5:**
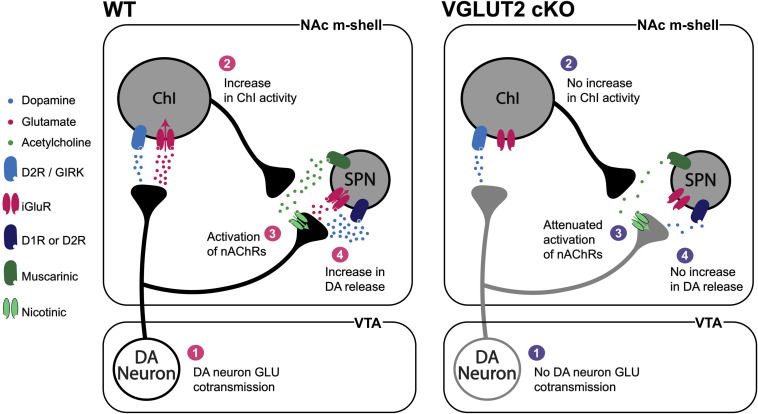
DA neuron GLU cotransmission circuit effects. DA neuron GLU cotransmission **(1)** increases ChI activity **(2)** and acetylcholine release that activates nAChRs on DA neuron terminals **(3)** to increase DA release **(4)**. In DA-neuron-specific *VGluT2* cKO mice **(1)** there would be no increase in ChI activity, **(2)** attenuated activation of nAChRs **(3)** and reduced DA release **(4)**. Vesicular synergy is not shown in this figure.

In the striatum, DA neurons make GLU connections preferentially to ChIs in the NAc m-shell and lateral dorsal striatum (Chuhma et al., 2014, 2018; Cai and Ford, 2018). ChIs are distributed throughout the striatum with widespread axonal arborizations. Most striatal neurons express acetylcholine receptors, particularly on their presynaptic terminals (Lim et al., 2014; Ztaou and Amalric, 2019). This points to widespread effects of DA neuron GLU cotransmission on striatal circuits via modulation of ChI activity (Stocco, 2012; Zhang and Cragg, 2017; Assous and Tepper, 2019). DA neuron GLU cotransmission can also exert positive feedback on DA neuron transmission via presynaptic nicotinic acetylcholine receptors (nAChRs) (Figure 5). In the m-shell, DA neuron GLU cotransmission activates ChIs directly with short latency (Chuhma et al., 2014; Mingote et al., 2017), potentially inducing synchronized activation of ChIs (Mingote et al., 2019). Increased ChI activity may then activate nAChRs on DA neuron terminals resulting in an increase in DA release (Cachope et al., 2012; Threlfell et al., 2012), forming a positive feedback loop. Lack of DA neuron GLU cotransmission in *DAT^*Cre*^;VGluT2^*flox/flox*^* cKO mice disrupts this loop; it also reduces DA release in the striatum, in line with disrupted vesicular synergy (Stuber et al., 2010; Alsiö et al., 2011).

Dopamine neuron GLU cotransmission appears to regulate activity in multiple brain regions. *DAT^*Cre*^;VGluT2^*flox/flox*^* cKO mice have widespread alterations in immediate early genes *c-fos* and *Nur77* in striatal subregions (Alsiö et al., 2011). Circuit-level alterations are also shown by an increase in AMPA/NMDA ratio in D1-receptor expressing SPNs in the NAc in tamoxifen-inducible DA-neuron-specific *VGluT2* cKO (*DAT^*Cre–ERT2*^;VGluT2^*flox/flox*^*) mice, in which *VGluT2* is conditionally excised from DA neurons in adulthood (Papathanou et al., 2018). In acute hippocampal slices, local field potential recordings revealed *TH^*IRES–Cre*^;VGluT2^*flox/flox*^* cKO mice had fewer kainate-induced gamma oscillations and more epileptic activity than controls (Nordenankar et al., 2015); suggesting network-wide effects that may alter excitation/inhibition balance involving multiple brain regions.

## What Are the Developmental Roles of DA Neuron GLU Cotransmission?

### Embryonic Differentiation of DA Neurons and Development of VGluT2 Expression

During development most, if not all, DA neurons in the ventral midbrain express *VGluT2*, and a substantial portion continue to do so in adulthood (Wallén-Mackenzie et al., 2006; Dal Bo et al., 2008; Birgner et al., 2010; Fortin et al., 2012; Trudeau et al., 2014; Steinkellner et al., 2018; Bimpisidis and Wallén-Mackenzie, 2019; Dumas and Wallén-Mackenzie, 2019; Kouwenhoven et al., 2020; Table 1). Embryonic cell-fate labeling shows that >90% of DA neurons in the VTA and SN in adult mice expressed *VGluT2* during development (Steinkellner et al., 2018; Kouwenhoven et al., 2020; Fougère et al., 2021).

In the medial VTA, where most DA-GLU neurons are located, DA neuron differentiation is directed by zinc finger transcription factor Gli2 (Kabanova et al., 2015). Gli2 mediates sonic hedgehog (Shh)-induced formation of DA neuron progenitor cells around embryonic day (E) 9. Conditional knockout (cKO) of Gli2 during this period in *En1^*Cre/+*^;Gli2^*zfd/flox*^* (termed *Gli2^Δ^^*Mb*^*^>^*^*E9.0*^*) cKO mice reduced the number of TH^+^ neurons by about 50% and TH^+^/VGLUT2^+^ neurons by about 70%, while the number of VGLUT2-only (i.e., TH^–^/VGLUT2^+^) neurons is unaffected (Kabanova et al., 2015). The decrease in TH^+^/VGLUT2^+^ DA neurons leads to a significant reduction of DA neuron GLU cotransmission to inhibitory interneurons in the PFC (Kabanova et al., 2015). Remarkably, Shh continues to provide trophic support to DA neurons in adulthood, as DA-neuron-specific Shh cKO (*Shh^*nLZC/C*^;DAT^*Cre*^*) accelerates DA neuron degeneration via failure of reciprocal trophic support (Gonzalez-Reyes et al., 2012).

In addition to being the vesicular glutamate transporter subtype preferentially expressed in DA neurons, *VGluT2* is also the predominant subtype expressed in the embryonic brain (Boulland et al., 2004). *VGluT2* null mice (*VGluT2*^*flox/flox;PCre*^) die shortly after birth due to the role of VGLUT2 in brainstem respiratory central pattern generators (Moechars et al., 2006; Wallén-Mackenzie et al., 2006). DA-neuron-specific *VGluT2* cKO, driven by either *DAT*^*Cre*^ or *TH*^*Cre*^ transgenes in *VGluT2*^*flox/flox*^ mice, is not lethal. However, the *VGluT2* cKO affects DA neuron survival, maturation (including projections and formation of connections), and response to injury (Dal Bo et al., 2008; Bérubé-Carrière et al., 2009; Fortin et al., 2012; Shen et al., 2018; Steinkellner et al., 2018; Kouwenhoven et al., 2020). Since *VGluT2* expression in nascent DA neurons is detected around E10, prior to expression of DA neuron markers (Dumas and Wallén-Mackenzie, 2019), even *DAT^*Cre*^;VGluT2^*flox/flox*^* and *TH^*Cre*^;VGluT2^*flox/flox*^* cKO mice likely express *VGluT2* in DA neurons transiently. *DAT* expression starts at E14 and Cre-dependent recombination in *DAT*^*Cre*^ mice is clearly observed at E17 (Bäckman et al., 2006), indicating that Cre-dependent *VGluT2* excision occurs in late embryonic life. TH expression begins before this, as shown by TH^+^/VGLUT2^+^ neurons detected during E11.5–12.5 (Birgner et al., 2010; Nordenankar et al., 2015). Thus, it is important to note that findings from studies using *TH^*Cre*^;VGluT2^*flox/flox*^* cKO mice represent an earlier loss of VGLUT2 in DA neurons during embryonic development whereas *DAT^*Cre*^;VGluT2^*flox/flox*^* cKO mice reflect the loss of VGLUT2 function in DA neurons in the early postnatal period.

### Regulation of Maturation and Growth

Dopamine neurons in *DAT^*Cre*^;VGluT2^*flox/flox*^* cKO mice have smaller soma size, shorter axonal lengths and reduced neurite complexity (Fortin et al., 2012). Although there were no apparent changes in the configuration of the medial forebrain bundle, the total number of TH^+^ neurons are reduced by ∼25% in the VTA and ∼20% in the SNc (Fortin et al., 2012). There are significant reductions in TH^+^ axon density and DA release, measured with cyclic voltammetry, in the NAc shell, but not in the NAc core (Fortin et al., 2012), consistent with the more prominent GLU cotransmission in the NAc shell. Expression of DA receptors was increased in both the dorsal and ventral striatum in *DAT^*Cre*^;VGluT2^*flox/flox*^* cKO mice, further suggesting a role for DA neuron GLU cotransmission in the establishment of meso-striatal projections (Alsiö et al., 2011).

In co-cultures of DA and GABA neurons, only ∼20% of TH^+^ neurons coexpress *VGluT2*, whereas in pure DA neuron cultures ∼50% of TH^+^ neurons coexpress *VGluT2* (Mendez et al., 2008b). GABA did *not* reduce TH^+^/VGLUT2^+^ co-labeling in DA neuron culture, suggesting that a contact-dependent mechanism is required for downregulation of *VGluT2* expression (Mendez et al., 2008b). Quinolinic acid lesions of the medial dorsal striatum led to increased *VGluT2* expression in midbrain DA neurons (Mendez et al., 2008b). This could be a consequence of lost neurotrophic support from postsynaptic targets, or lack of afferent inputs to midbrain DA neurons. A more recent study showed that co-culture of DA neurons with dorsal striatal neurons reduced *VGluT2* mRNA expression, whereas co-culture of DA neurons with ventral striatal neurons increased *VGluT2* expression (Fortin et al., 2019). These findings suggest further that striatal neurons exert trophic effects on *VGluT2* expression in midbrain DA neurons. Overall, both pre and postsynaptic mechanisms appear to be important for growth and survival of DA-GLU neurons.

## How Are DA-GLU Neurons Affected by DA Neuron Toxins?

DA-GLU neurons appear to be less vulnerable to the DA neuron toxins 6-hydroxydopamine (6-OHDA) and 1-methyl-4-phenyl-1,2,3,6-tetrahydropyridine (MPTP) (Table 2). Intraventricular 6-OHDA injections in juvenile and adult rats increase the proportion of TH^+^/VGLUT2^+^ neurons among TH^+^ neurons in the VTA (Dal Bo et al., 2008; Bérubé-Carrière et al., 2009). 6-OHDA injections in the dorsal striatum increase the proportion of TH^+^/VGLUT2^+^ neurons in the SN (Steinkellner et al., 2018), and TH^+^/VGLUT2^+^ axon terminals in the NAc (Bérubé-Carrière et al., 2009). This increased ratio of TH^+^/VGLUT2^+^ neurons in ventral midbrain DA neurons after toxin exposure could be due to re-expression of *VGluT2* in the surviving TH^+^/VGLUT2^–^ neurons (i.e., ‘neurotransmitter switching,’ see Spitzer, 2015 for review), or reduced susceptibility of TH^+^/VGLUT2^+^ neurons. Thus, an increase of TH^+^/VGLUT2^+^ projections in the striatum could be due to new projections of VTA TH^+^/VGLUT2^+^ neurons compensating for the loss of SN TH^+^/VGLUT2^+^ neurons, or SN TH^+^/VGLUT2^–^ neurons switching to TH^+^/VGLUT2^+^, resulting in an increase in the number of DA-GLU neurons. In mouse SN DA neuron culture, 1-methyl-4-phenylpyridinium (MPP+) exposure increases *VGluT2* copy number per cell, while TH copy number per cell is reduced (Kouwenhoven et al., 2020). This suggests that cellular stress drives neurotransmitter switching and similar mechanisms may be activated in surviving DA neurons after toxin exposure.

**TABLE 2:** Effect of toxins on DA neuron GLU cotransmission.

Species and Age	Genotype	Method	Toxin	TH^+^VGLUT2^+^/Total TH^+^%		Effect on DA neurons	Citation
				VTA	SN		
Rat P15	WT	ISH	**Vehicle**	7.1	0.7	In NAc% TH^+^/VGLUT2^+^ axon terminals higher in 6-OHDA group (37.4%) *vs.* vehicle (28.2%)	Dal Bo et al., 2008
			**6-OHDA** Intraventricular on P4 (perfusion 11d later)	26	0.0		
Mouse P52	WT	IHC	**Vehicle**	15	9.0	Increased loss of SN TH^+^ neurons	Steinkellner et al., 2018
			**6-OHDA** Dorsal striatum on P42 (perfusion 10d later)	19	21		
	*DAT^*IRES–Cre/+*^; VGluT2*^*flox/+*^ or *DAT^*IRES–Cre/+*^; VGluT2*^*flox/flox*^	IHC	**6-OHDA** Dorsal striatum on P42 (perfusion 10d later)	−		Increased loss of SN TH^+^ neurons, significantly more in cKO mice	
Mouse P8–12 weeks	*DAT^*IRES–Cre/+*^; VGluT2*^*flox/+*^ or *DAT^*IRES–Cre/+*^; VGluT2*^*flox/flox*^	IHC	**Acute MPTP** 15 mg/kg i.p. × 4 2 h apart, same day. (perfusion 7 h later)	−		Increased loss of midbrain TH^+^ neurons in cKO mice	
			**Chronic MPTP** 30 mg/kg i.p. × 5 days (perfusion 21 days later)	−		Increased loss of midbrain TH^+^ neurons in cKO mice	
Mouse P8–12 weeks	*DAT^*IRES–Cre/+*^; VGluT2*^*flox/+*^	IHC	**Vehicle**	15	20	Increased loss of VTA and SN TH^+^ neurons in cKO mice. Reduced TH and DAT throughout striatum. Viral rescue of *VGluT2* in DA neurons slightly attenuated reduction	Shen et al., 2018
			**Acute MPTP** 18 mg/kg; i.p. × 4 2 h apart, same day. (perfusion 14 days later)	35	45		
	*DAT^*IRES–Cre/+*^; VGluT2*^*flox/flox*^		**Acute MPTP** 18 mg/kg; i.p. × 4 2 h apart, same day. (perfusion 14 days later)	–	–		
Mouse > P60	*DAT^*IRES–Cre/+*^; VGluT2*^*flox/flox*^		**Acute 6-OHDA** Dorsal striatum (perfusion 7 weeks later)	-	-	Impaired striatal re-innervation post-6-OHDA in cKO mice.	Kouwenhoven et al., 2020
Rat P90	WT	ISH	**Artificial cerebrospinal fluid**	2.4	0.3	In NAc% of TH^+^/VGLUT2^+^ axon terminals higher with 6-OHDA (0.05%) *vs.* vehicle (0%)	Bérubé-Carrière et al., 2009
			**6-OHDA** Intraventricular on P4 (perfusion 11 days later)	7.2	0.0		

SN DA neurons in *DAT^*IRES–Cre/+*^;VGluT2^*flox/flox*^* cKO mice are more vulnerable to 6-OHDA and MPTP than those in *DAT^*IRES–Cre/+*^;VGluT2^*flox/+*^* conditional heterozygous (cHET) control mice (Shen et al., 2018; Steinkellner et al., 2018; Kouwenhoven et al., 2020). *DAT^*IRES–Cre/+*^;VGluT2^*flox/flox*^* cKO mice, compared to cHET mice, have reduced levels of brain derived neurotrophic factor and its receptor TrkB in VTA and SN DA neurons, and are more vulnerable to MPTP (Shen et al., 2018). Viral rescue of *VGluT2* expression with an AAV-DIO-*VGluT2* vector in DA neurons of *DAT^*IRES–Cre*^;VGluT2^*flox/flox*^* cKO mice recovered brain derived neurotrophic factor/TrkB expression and thereby attenuated MPTP-induced DA neuron toxicity. MPTP-induced expression of proapoptotic marker BAX in the midbrain is not different between *DAT^*IRES–Cre/+*^;VGluT2^*flox/flox*^* cKO mice and cHET control mice, suggesting that a neuroprotective function of VGLUT2 is not related to production of proapoptotic/antiapoptotic factors (Shen et al., 2018). Thus, *VGluT2* expression appears to be neuroprotective via neurotrophic signaling rather than an anti-apoptotic mechanism. However, VGLUT2 appears not to have a purely protective effect as overexpression of *VGluT2* is neurotoxic in both flies and mice, leading to upregulation of markers of apoptosis and inflammatory gliosis (Steinkellner et al., 2018).

## What Are the Behavioral Roles of DA Neuron GLU Cotransmission?

In this section we have parsed pre-clinical behavioral findings from studies of DA neuron GLU cotransmission along the Research Domain Criteria delineated by the National Institute of Mental Health (Table 3–7). The Research Domain Criteria were constructed to provide a research framework for mental disorders based on multiple levels, from genomics to behaviors, organized around major divisions called *domains* and subdivisions called *constructs*, meant to encapsulate different aspects that model human functioning in areas related to emotion, cognition and behavior (Insel, 2014). Using this format facilitates comparisons across studies and species.

**TABLE 3:** Positive valence systems: reward responsiveness construct.

Paradigm		Manipulation	Behavioral Result	Citation
*Cocaine, acute response*	20 mg/kg i.p.	*DAT^*Cre*^;VGluT2^*flox/flox*^*	**Decreased** response	Hnasko et al., 2010
	10 mg/kg i.p.	*DAT^*Cre*^;VGluT2^*flox/flox*^*	**Decreased** response	Fortin et al., 2012
	20 mg/kg i.p.	Heterologous *VGluT2* overexpression. Unilateral SNc *DAT*^*Cre*^	**Decreased** response	Steinkellner et al., 2018
*Cocaine sensitization*	5 days of daily injections (20 mg/kg i.p) and re-test 72 h later	*DAT^*Cre*^;VGluT2^*flox/flox*^*	**Intact** sensitization (cKO mice steadily increased responses over days 1–4, but at **lower** levels. By day 5 and on challenge cKO mice had similar responses.	Hnasko et al., 2010
	5 days of daily injections (20 mg/kg i.p.). No re-test	*DAT^*Cre*^;VGluT2^*flox/flox*^; DRD1-EGFP*	Intact sensitization. However, on day 5, cKO mice had **less distance** traveled.	Papathanou et al., 2018
		*DAT^*Cre–ERT2*^; VGluT2^*flox/flox*^; DRD1-EGFP* Tamoxifen 2 mg i.p. daily × 5 days at P8–9 weeks.	Intact sensitization. Though, overall, **less distance** traveled in tamoxifen-treated group.	
*Amphetamine, acute response*	1.5, 3.0, and 5.0 mg/kg i.p.	*DAT^*Cre*^;VGluT2^*flox/flox*^*	Overall activity of cKO **lower** than cHET, though total activity and rearing rose with increased doses.	Birgner et al., 2010
	0.75 mg/kg i.p.	*DAT^*Cre*^;VGluT2^*flox/flox*^*	**Decreased** response.	Fortin et al., 2012
	1.5 mg/kg i.p.	*TH^*IRES–Cre*^;VGluT2^*flox/flox*^*	**Unaltered**.	Nordenankar et al., 2015
	3.0 and 5.0 mg/kg i.p.	*DAT^*IRES–Cre*^;Gls1^*flox/+*^*	**Unaltered**.	Mingote et al., 2017
	3.0 mg/kg i.p.	Heterologous *VGluT2* overexpression. Unilateral SNc *DAT*^*Cre*^	**Decreased** response.	Steinkellner et al., 2018
*Amphetamine sensitization*	Five daily injections of 2.5 mg/kg i.p. Challenge to same dose 2 weeks later.	*DAT^*IRES–Cre*^;Gls1^*flox/+*^*	**No sensitization** over 5 days. Blunted response to challenge at 2 weeks.	Mingote et al., 2017
	Four daily injections of 3.0 mg/kg i.p. Challenge to same dose 2 weeks later	*DAT^*Cre*^;VGluT2^*flox/flox*^*	**No sensitization** over 4 days. Blunted response to challenge at 2 weeks. Repeated protocol 1 week later with 2 challenges, no sensitization.	Papathanou et al., 2018
		*DAT^*Cre–ERT2*^;VGluT2^*flox/flox*^; DRD1-EGFP* Tamoxifen 2mg i.p. daily x 5d at P8-9w.	Both groups showed an increase in AMPH-induced locomotion, **no difference** between genotypes.	

**TABLE 4:** Positive valence systems: reward learning construct.

Paradigm		Manipulation	Behavioral Result	Citation
*Cocaine conditioned place preference*	5 mg/kg s.c. for 3 days	*DAT^*Cre*^;VGluT2^*flox/flox*^*	**Unaltered**	Hnasko et al., 2010
*Cocaine IV Self-administration*	0.0625, 0.125, and 1.0 mg/kg infusion	*DAT^*Cre*^;VGluT2^*flox/flox*^*	**Enhanced** at low dose; **unaltered** at higher doses	Alsiö et al., 2011
*Cocaine-seeking to drug-paired cues*		*DAT^*Cre*^;VGluT2^*flox/flox*^*	**Increased** by 76%	
*Operant conditioning high-sucrose food*		*DAT^*Cre*^;VGluT2^*flox/flox*^*	**Enhanced**	
*Intracranial self-optogenetic-stimulation VTA TH^+^ neurons*		*DAT^*IRES–Cre/+*^;VGluT2^*flox/flox*^* Viral DIO-*ChR2* into VTA	**Slight impairment** with 32 mW/3 ms stimulation. **No difference** during 1st five sessions with 8 mW/1 ms stimulation.	Wang et al., 2017
*Conditioned Place Preference to VTA TH^+^ neuron optogenetic-stimulation*		*DAT^*IRES–Cre/+*^;VGluT2^*flox/flox*^* Viral DIO-*ChR2* into VTA	**No difference**.	
*Intracranial self-stimulation of NAc m-shell*		*VGluT2^*Cre*^;TH^*flox/flox*^* Viral DIO-*ChR2* into VTA	**No difference**. Equivalent preference for nosepoke hole coupled to optogenetic stimulation.	Zell et al., 2020
*Intracranial self-stimulation of VTA*				
*Real-time place preference of NAc m-shell*			**No difference**. Loss of DA from VGLUT2^+^ neurons did not alter response (avoidance of 40 Hz optogenetic stimulation)	
*Real-time place preference of VTA*				
*Intracranial self-stimulation of NAc m-shell*		*VGluT2*^*Cre*^ Viral DIO-*ChR2* and Viral FLEX-SaCas9-sg*Th* into VTA	**No difference**. Equivalent preference for nosepoke hole coupled to optogenetic stimulation	
*Intracranial self-stimulation of VTA*				
*Real-time place preference of NAc m-shell*			**No difference**. Loss of DA from VGLUT2^+^ neurons did not alter response (avoidance of 40 Hz optogenetic stimulation)	
*Real-time place preference of VTA*				

**TABLE 5:** Cognitive control systems.

Paradigm	Manipulation	Behavioral Result	Citation
*Radial arm maze*	*TH^*IRES–Cre*^;VGluT2^*flox/flo*^*^*x*^	**Impaired:** cKO mice made more reference memory errors.	Nordenankar et al., 2015
*Latent inhibition*	*DAT^*IRES–Cre*^;Gls1^*flox/+*^*	**Potentiated:** sub-threshold pre-exposure to tone sufficient to induce latent inhibition in cHET mice.	Mingote et al., 2017

**TABLE 6:** Negative valence systems.

Paradigm	Manipulation	Behavioral Result	Citation
***Construct: Acute threat (“Fear”)***			
*Elevated plus maze*	*DAT^*Cre*^;VGluT2^*flox/flox*^*	**Increased** latency to start	Birgner et al., 2010
	*TH^*IRES–Cre*^;VGluT2^*flox/flox*^*	**Normal**	Nordenankar et al., 2015
	*DAT^*IRES–Cre*^;Gls1^*flox/+*^*	**Normal**	Mingote et al., 2017
	*DAT^*IRES–Cre*^;VGluT2^*flox/flox*^*	**Increased** anxiety after MPTP administration	Shen et al., 2018
*Fear conditioning*	*DAT^*IRES–Cre*^;Gls1^*flox/+*^*	**Normal**	Mingote et al., 2017
*Open field test*	*DAT^*Cre*^;VGluT2^*flox/flox*^*	**Decreased** time in the central circle of the open field	Birgner et al., 2010
	*DAT^*IRES–Cre*^;Gls1^*flox/+*^*	**Normal**	Mingote et al., 2017
***Construct: Sustained threat***			
*Forced swim test*	*DAT^*Cre*^;VGluT2^*flox/flox*^*	**Normal**	Birgner et al., 2010
	*DAT^*Cre*^;VGluT2^*flox/flox*^*	**Normal** (though decreased latency to immobilization on Day 1)	Fortin et al., 2012
	*TH^*IRES–Cre*^;VGluT2^*flox/flox*^*	**Normal**	Nordenankar et al., 2015

**TABLE 7:** Motor control systems.

Paradigm	Manipulation	Behavioral Result	Citation
*Locomotor activity*	*DAT^*Cre*^;VGluT2^*flox/flox*^*	**No difference** in novelty-associated locomotion over 4 h or total locomotion across 3 days	Hnasko et al., 2010
	*DAT^*Cre*^;VGluT2^*flox/flox*^*	**No difference** in locomotion or rearing activity in novel environment; decreased horizontal activity	Fortin et al., 2012
	*TH^*IRES–Cre*^;VGluT2^*flox/flox*^*	**Normal**	Nordenankar et al., 2015
	*DAT^*IRES–Cre*^;Gls1^*flox/+*^*	**Normal**	Mingote et al., 2017
	Heterozygous *VGluT2* over-expression unilateral SNc *DAT*^*Cre*^	**Significantly reduced** spontaneous locomotor activity	Steinkellner et al., 2018
	*DAT^*IRES–Cre*^;VGluT2^*flox/flox*^*	MPTP induced a **significant reduction** in vertical activity. *Viral rescue of VGluT2 in DA neurons attenuated these reductions.*	Shen et al., 2018
*Accelerating rotarod*	*DAT^*Cre*^;VGluT2^*flox/flox*^*	**Normal**	Birgner et al., 2010
	*DAT^*Cre*^;VGluT2^*flox/flox*^*	**Normal** across 5 days	Hnasko et al., 2010
	*DAT^*Cre*^;VGluT2^*flox/flox*^*	**Impaired** (significant decrease in distance day 1, speed/latency to fall both days)	Fortin et al., 2012
	*DAT^*IRES–Cre*^;Gls1^*flox/+*^*	**Normal** across 3 days	Mingote et al., 2017
	*DAT^*IRES–Cre*^;VGluT2^*flox/flox*^*	**No difference** in MPTP-induced deficits	Shen et al., 2018
*Beam walk*	*DAT^*Cre*^;VGluT2^*flox/flox*^*	**Normal**	Birgner et al., 2010
*Parallel rod floor*	*DAT^*IRES–Cre*^;VGluT2^*flox/flox*^*	MPTP-induced **deficits pronounced** in cKO mice. *Deficits were restored by viral rescue of VGluT2 expression in DA neurons.*	Shen et al., 2018

### Positive Valence Systems

Within the Positive Valence Systems domain, DA neuron GLU cotransmission affects two constructs: reward-responsiveness (Table 3) and reward learning (Table 4). Disruption of DA neuron GLU cotransmission in *DAT^*Cre*^;VGluT2^*flox/flox*^* cKO mice blunts acute responses to psychostimulants (Birgner et al., 2010; Hnasko et al., 2010; Fortin et al., 2012; Steinkellner et al., 2018). Although *DAT^*Cre*^;VGluT2^*flox/flox*^* cKO mice were initially hyporesponsive to doses of cocaine, they still showed sensitization (Hnasko et al., 2010) — a measure of increasing reward-responsiveness to repeated exposures to the same dose, which models pathologic incentive motivation in addiction (Robinson and Berridge, 2008). Conversely, cHET of GLU recycling enzyme glutaminase (GLS1) in DA neurons did not affect acute responses to amphetamine, but did diminish sensitization and blunted responses to subsequent challenge doses (Mingote et al., 2017). Even when initial responses are intact, impaired DA neuron GLU cotransmission still disrupts reward responsiveness. Since reduced GLU cotransmission does not affect motor control or negative valence systems (see below), the blunted reward responsiveness is not secondary to motor or emotional impairment.

Cocaine-seeking induced by drug-paired cues and cocaine intravenous self-administration are enhanced in *DAT^*Cre*^;VGluT2^*flox/flox*^* in cKO mice (Alsiö et al., 2011). Operant conditioning for high-sucrose food is also enhanced in *DAT^*Cre*^;VGluT2^*flox/flox*^* cKO mice, showing that DA neuron GLU cotransmission modulates intensity of responses not only to psychostimulants, but also to natural rewards (Alsiö et al., 2011). *DAT^*IRES–Cre*^;VGluT2^*flox/flox*^* cKO mice showed reduced progressive intracranial optogenetic self-stimulation of VTA TH^+^ neurons, supporting the hypothesis that DA neuron GLU cotransmission regulates the magnitude of operant behaviors (Wang et al., 2017). Although GLU released from DA neurons may not be critical for the acquisition of conditioned reinforcement, its loss nonetheless affects positive valence systems. For example, *VGluT2^*Cre*^;TH^*flox/flox*^* cKO mice, which have *TH* excised from VGLUT2^+^ DA neurons (i.e., DA neurons with blunted DA transmission but intact GLU cotransmission), optogenetic stimulation of *VGluT2*^*Cre/AAV–DIO–ChR2*^ VTA neurons was sufficient to reinforce behavior (Zell et al., 2020). Although this study did not discriminate contributions of GLU-only (non-DAergic) neurons and GLU cotransmission from DA-GLU neurons, GLU cotransmission from DA-GLU neurons presumably contributes to DA-independent positive reinforcement.

The only DA-neuron-specific *VGluT2* cKO study without an impaired response to acute psychostimulants used a *TH*^*IRES–Cre*^ transgene instead of a *DAT*^*Cre*^ or *DAT*^*IRES–Cre*^ transgene to establish the DA-neuron-specific *VGluT2* cKO (Nordenankar et al., 2015). Subsequent reviews have cautioned about comparisons between *TH*^*Cre*^ and *DAT*^*Cre*^ induced conditional gene expression (Pupe and Wallén-Mackenzie, 2015; Stuber et al., 2015; Lammel et al., 2015; Buck et al., 2020; Fischer et al., 2020). Briefly, *TH*^*Cre*^ mice cause more developmental effects than *DAT*^*Cre*^ mice, because *TH* expression begins earlier in development than *DAT* (see above), and is more widespread and ectopic (i.e., neurons that are positive for *TH* mRNA but not TH protein) (Di Porzio et al., 1990). Although, *DAT*^*Cre*^ mice also show off-target recombination in a subset of DAT-negative neurons in particular limbic areas (Papathanou et al., 2019). Also, because TH is part of the synthetic pathway of norepinephrine, norepinephrine neurons will be affected in *TH*^*Cre*^ mice as well. It should be noted that intensity of responses to psychostimulants can also be affected by background strain, e.g., *C57BL/6J* mice show greater responses than *129S2/SvHsd* mice (Chen et al., 2007). Although the background strain issue is partly mitigated by use of littermate controls, difference in background strains must be considered when comparing studies (Crawley et al., 1997; Bailey et al., 2006; Linder, 2006, 2001; Yoshiki and Moriwaki, 2006).

Behavioral studies using cKO mice with *DAT* or *TH* promoters to drive Cre recombinase to excise floxed *VGluT2* from DA neurons must be interpreted with caution, because effects seen in adulthood can be caused by developmental derangements *and/or* effects of diminished GLU cotransmission in adulthood. Both *DAT* and *TH* are expressed during embryogenesis (Di Porzio et al., 1990; Bäckman et al., 2006), thus, *DAT^*Cre*^;VGluT2^*flox/flox*^* and *TH^*Cre*^;VGluT2^*flox/flox*^* cKO mice lose VGLUT2 function in DA neurons in early life (see above). For example, *DAT^*Cre*^;VGluT2^*flox/flox*^* cKO mice show impaired responses to psychostimulants, and have reduced TH^+^ neuron numbers, thus the impaired responses to psychostimulants could be due to lack of DA neuron GLU cotransmission in adulthood and/or reduced TH^+^ neurons (Birgner et al., 2010; Fortin et al., 2012). Of note, *DAT^*IRES–Cre*^;Gls1^*flox/+*^* cHET mice also have impaired responses to psychostimulants, *despite* unaffected DA neuron number or DA release (Mingote et al., 2017). To further circumvent issues related to developmental alterations, Papathanou and colleagues knocked out *VGluT2* from DA neurons in adulthood using tamoxifen-inducible DA-neuron-specific *VGluT2* cKO (*DAT^*Cre–ERT2*^;VGluT2^*flox/flox*^*) mice (Papathanou et al., 2018). Control *DAT^*Cre*^;VGluT2^*flox/flox*^* cKO mice showed blunted sensitization to cocaine and amphetamine, in agreement with previous studies (Hnasko et al., 2010; Fortin et al., 2012; Mingote et al., 2017), whereas *DAT^*Cre–ERT2*^;VGluT2^*flox/flox*^* cKO mice given tamoxifen at 8–9 weeks of age did not show psychostimulant-induced hyperlocomotion (Papathanou et al., 2018), thus demonstrating that DA-neuron-specific *VGluT2* expression in adulthood is necessary for full psychostimulant responsivity. A potential confound is that all mice receiving tamoxifen showed blunted responses to psychostimulants – regardless of genotype (i.e., both *DAT^*Cre–ERT2*^;VGluT2^*flox/flox*^* cKO and *DAT^*Cre–ERT2*^;VGluT2^*flox/+*^* cHET). These blunted responses to psychostimulants could be due to tamoxifen itself, which impairs locomotor responses to amphetamine, even if tamoxifen is not given on the day of locomotor testing (Mikelman et al., 2018). Nonetheless, this suggests that DA neuron GLU cotransmission later in life still mediates psychostimulant responses, but perhaps less so than estimated from observations in *DAT^*Cre*^;VGluT2^*flox/flox*^* and *TH^*Cre*^;VGluT2^*flox/flox*^* cKO mice.

### Cognitive Control

Roles for DA neuron GLU cotransmission in the cognitive control domain have been studied with latent inhibition and tests of spatial working memory (Table 5). Latent inhibition is a testable cognitive behavior with clinical relevance to schizophrenia, observed in both rodent models and in clinical studies (Gaisler-Salomon et al., 2009; Weiner and Arad, 2009). Latent inhibition assesses how pre-exposure to a conditioned stimulus (CS; typically, a tone) prevents formation of an association between that CS and an unconditioned stimulus (US; typically, a shock). In mice, testing for latent inhibition has three phases. First, *the CS-only pre-exposure phase*, all mice are placed in a chamber but only the experimental group is exposed several times to a tone, whereas the control group is not. Second, *the CS-US pairing phase*, both groups of mice are placed in the testing chamber and receive a footshock paired with the tone. Last, *the CS-only test phase*, all mice are exposed to the tone and freezing behaviors are measured. Sufficient pre-exposure to the tone reduces freezing during the CS-only test phase, despite the temporal delay between pre-exposure and test (*latent inhibition*). *DAT^*IRES–Cre*^;Gls1^*flox/+*^* cHET mice showed an enhanced latent inhibition, i.e., an enhanced ability to discriminate cue saliency (Mingote et al., 2017), suggesting that abrogated GLU release from DA neurons facilitates cognitive function.

*TH^*IRES–Cre*^;VGluT2^*flox/flox*^* cKO mice have impaired learning a radial arm maze, a task used to assess spatial working memory (Nordenankar et al., 2015). Although *TH^*IRES–Cre*^;VGluT2^*flox/flox*^* cKO mice were still able to learn the task, they took significantly longer and made more reference memory errors, but not working memory errors, than *TH^*IRES–Cre*^;VGluT2^*flox/+*^* cHET controls (Nordenankar et al., 2015). Reference memory errors are thought to reflect hippocampal deficits, whereas working memory errors reflect impairments in frontal cortical networks (Yoon et al., 2008). Lack of DA neuron GLU cotransmission appears to impair hippocampal reference memory, suggesting that intact cotransmission may facilitate spatial reasoning beyond simply improving attention. *Gli2^Δ^^*Mb*^*^>^*^*E9.0*^* cKO also results in a substantial reduction in medial VTA TH^+^/VGLUT2^+^ neurons and increases perseverative behavior on the five-choice serial reaction time task, suggesting impaired visuospatial attention and motor impulsivity (Kabanova et al., 2015). However, the contribution of mesocortical GLU-only neurons, which are also reduced by *Gli2* cKO in DA neurons, cannot be excluded. Again, since reduced GLU cotransmission does not appear to affect motor control or negative valence systems (see below), the effects on cognitive control are not secondary to motor or emotional impairment.

### Negative Valence Systems

Behaviors related to acute and sustained threats are largely unaffected by impaired DA neuron GLU cotransmission (Birgner et al., 2010; Fortin et al., 2012; Nordenankar et al., 2015; Mingote et al., 2017; Table 6). Standard tests of anxiety, such as the elevated-plus maze and open field test, are mostly unaffected in *DAT^*Cre*^;VGluT2^*flox/flox*^* cKO mice and *TH^*IRES–Cre*^;VGluT2^*flox/flox*^* cKO mice (Birgner et al., 2010; Nordenankar et al., 2015); however, after MPTP administration, *DAT^*IRES–Cre*^;VGluT2^*flox/flox*^* showed increased anxiety on the elevated-plus maze (Shen et al., 2018). Similarly, freezing in a fear-conditioning paradigm did not differ in *DAT^*IRES–Cre*^;Gls1^*flox/+*^* cHET mice (Mingote et al., 2017). Performance on the forced-swim test, a measure of a depressive-like phenotype, is largely unchanged in *DAT^*Cre*^;VGluT2^*flox/flox*^* cKO mice and *TH^*IRES–Cre*^;VGluT2^*flox/flox*^* cKO mice (Birgner et al., 2010; Nordenankar et al., 2015), though one study showed a decreased latency to immobilization on day one but not on day two in *DAT^*Cre*^;VGluT2^*flox/flox*^* cKO mice (Fortin et al., 2012).

### Motor Control

Loss or decrease of DA neuron GLU cotransmission, whether in *DAT^*Cre*^;VGluT2^*flox/flox*^* cKO mice, *TH^*IRES–Cre*^;VGluT2^*flox/flox*^* cKO mice or *DAT^*IRES–Cre*^;Gls1^*flox/+*^* cHET mice, does not alter basic motor and arousal function (Birgner et al., 2010; Hnasko et al., 2010; Fortin et al., 2012; Nordenankar et al., 2015; Mingote et al., 2017), with few exceptions in one study using *DAT^*IRES–Cre/+*^;VGluT2^*flox/flox*^* cKO mice (Steinkellner et al., 2018; Table 7). Gross locomotor function is normal in *DAT^*Cre*^;VGluT2^*flox/flox*^* cKO mice and *TH^*IRES–Cre*^;VGluT2^*flox/flox*^* cKO mice (Hnasko et al., 2010; Fortin et al., 2012; Nordenankar et al., 2015). Motor coordination tested with rotarod is normal in studies using both sexes of *DAT^*Cre*^;VGluT2^*flox/flox*^* cKO mice (Birgner et al., 2010; Hnasko et al., 2010), although one study using only *DAT^*Cre*^;VGluT2^*flox/flox*^* cKO male mice showed impairment (Fortin et al., 2012). It remains unresolved whether this reflects variation between studies or differential effects between males and females, as no female-only study has been performed. MPTP-induced motor impairments were more pronounced in *DAT^*IRES–Cre*^;VGluT2^*flox/flox*^* cKO mice, but restored by *VGluT2* viral rescue (Shen et al., 2018). The lack of change in motor control could be related to lesser DA neuron GLU cotransmission in the dorsal striatum, which is more associated with motor learning.

## Does DA Neuron GLU Cotransmission Have a Role in Human Disorders?

Understanding behavioral roles of DA-GLU neurons offers potential insight into human neuropsychiatric disorders. Interactions between DA and GLU figure prominently in neuropsychiatric disorders, and DA neuron GLU cotransmission is one of the points where DA and GLU interact.

### Substance Use Disorders/Addiction

In humans, post-mortem studies of cigarette smokers have demonstrated increased VTA *VGLUT2* (human gene) expression compared to healthy controls (Flatscher-Bader et al., 2008). Given that microarrays were performed specifically in the VTA, even though TH-VGLUT2 double-staining was not performed, it is likely some of the *VGLUT2* expressing neurons were DA neurons, suggesting that either increased cotransmission may be a risk factor for smoking or that smoking may alter *VGLUT2* expression in DA neurons. In mice, neonatal nicotine exposure increases numbers of DA-GLU neurons and nicotine preference in adulthood (Romoli et al., 2019). Selectively targeting DA neuron GLU cotransmission may thus serve as a potential treatment for addiction (Bimpisidis and Wallén-Mackenzie, 2019), especially psychostimulant use disorders perhaps by facilitating behavioral switching (Mingote et al., 2019). Further discussion about DA-GLU neurons and addiction is found in recent reviews (Trudeau et al., 2014; Steinkellner et al., 2018; Bimpisidis and Wallén-Mackenzie, 2019; Buck et al., 2020; Fischer et al., 2020).

### Psychotic Disorders

Both DA and GLU are implicated in the patho-etiology of schizophrenia by findings ranging from psychopharmacology, post-mortem analyses and *in vivo* brain imaging (for review see Howes et al., 2015). DA neuron GLU cotransmission serves as one potential point of confluence of DA and GLU actions (Chuhma et al., 2017).

One specific role of DA-GLU cotransmission is perhaps best demonstrated in studies of latent inhibition, which models cognitive impairments in schizophrenia, as well as in animal models (Weiner and Arad, 2009). Humans at high-risk for developing psychosis demonstrate deficits in latent inhibition, suggesting it is a cognitive marker of psychotic propensity, rather than a secondary effect of medication or a consequence of chronic schizophrenia (Kraus et al., 2016). As mentioned above, potentiation of latent inhibition in DA neuron *DAT^*IRES–Cre*^;Gls1^*flox/+*^* cHET mice (Mingote et al., 2017) emphasizes the therapeutic potential of reducing DA neuron GLU cotransmission.

### Parkinson Disease

The main motor symptoms of Parkinson Disease (PD) are primarily due to the loss of nigrostriatal DA neurons. A recent study found that following partial loss of DA inputs, DA-driven inhibition of cholinergic activity in the dorsomedial striatum is preserved due to reduced DA reuptake, while GLU co-release evoked excitation in the dorsolateral striatum is lost due to a downregulation of mGluR1 (Cai et al., 2021). Altered DA-acetylcholine interactions have been hypothesized to underpin some of the symptoms of PD (Ztaou and Amalric, 2019). Since DA neuron GLU cotransmission regulates ChI activity, elucidating mechanisms of this regulation may help delineate PD pathophysiology and therapeutics.

One of the most promising treatments for PD is stem cell implantation (Widner et al., 1992; Mendez et al., 2002, 2008a; Wijeyekoon and Barker, 2009). For successful implantation, it is crucial to choose DA neurons in the appropriate developmental stage to survive and form connections (Lindvall, 2012), which may benefit from appropriate *VGluT2* expression levels. For example, wildtype *VGluT2* expression appears to be neuroprotective to DA neurons in PD mouse models (Dal Bo et al., 2008; Bérubé-Carrière et al., 2009; Shen et al., 2018; Steinkellner et al., 2018; Kouwenhoven et al., 2020), though *VGluT2* overexpression appears to be neurotoxic to DA neurons (Steinkellner et al., 2018). Thus, determining a specific range of appropriate *VGluT2* expression levels to optimize survival may be an important consideration in transplantation protocols to treat PD.

## Potential Directions for Circuit-Based Pharmacotherapy

Given its involvement in circuitry underlying various neuropsychiatric disorders — ranging from schizophrenia, addiction, to PD — DA neuron GLU cotransmission is a considerable target of treatment for neuropsychiatric disorders. Refined molecular genetic manipulations can target discrete DA neuron subtypes, opening up new avenues for investigation and serving as proof-of-principle for future treatment of neuropsychiatric disorders.

One such approach is Genetic Pharmacotherapy, which is defined as the use of genetic interventions in mouse models to elucidate potential drug targets prior to the development of specific ligands (Gellman et al., 2011). This strategy enables the evaluation of therapeutic potential for target gene modification without costly and time-consuming development of specific ligands that may lack regional specificity and face issues regarding blood-brain barrier permeability. Genetic Pharmacotherapy achieves region-specific functional modulation by using molecular genetic techniques, such as conditional gene knockouts, to target neurons that express specific markers. This approach has already shown DA neuron GLU cotransmission as a viable target in schizophrenia treatment; DA neuron specific reduction of the GLU recycling enzyme GLS1 affects behaviors relevant to schizophrenia (Mingote et al., 2015b, 2017).

Furthermore, preclinical findings of neural function are applied to clinical trials using gene therapy with non-replicative, non-toxic viral vectors (for review see Lykken et al., 2018). Gene therapy requires characterization of specific circuits impacting a neuropsychiatric disorder, rather than pharmacologic targeting of specific, but widely distributed, cell-signaling receptors (Gordon, 2016). Additionally, because gene therapy can be brain-region specific, and even cell-type specific, it would presumably have less off-target effects compared to oral medications. DA neuron GLU cotransmission is an example of how a genetically distinct neuronal subpopulation affects phenotypes relevant to neuropsychiatric disorders, thus serving as a target for treatment development.

## Conclusion

Dopamine neurons capable of GLU cotransmission serve as an example of how a specific subset of neurons within a diverse neuronal population can have distinct functions. As the gap between bench and bedside narrows and therapeutic options widen, e.g., non-pharmacological interventions such as gene therapy with intersectional control, DA neuron GLU cotransmission may be targeted for treatment of neuropsychiatric disorders.

## Author Contributions

DE, SR, and NC: concept, design, and writing. SR and NC: supervision. DE: literature research. DE, LM, SM, LY, SZ, VV, SR, and NC: analysis, interpretation, and critical review. All authors agreed to be accountable for the content of the work.

## Conflict of Interest

The authors declare that the research was conducted in the absence of any commercial or financial relationships that could be construed as a potential conflict of interest.

## Abbreviations

6-OHDA: 6-hydroxydopamine
BLA: basolateral amygdala
CeA: central nucleus of the amygdala
ChI: cholinergic interneuron
cHET: conditional heterozygous
CingC: cingulate cortex
cKO: conditional knockout
CLi: central linear nucleus
CS: conditioned stimulus
DA: dopamine
DAT: dopamine transporter
E#: embryonic day
EGFP: enhanced green fluorescent protein
EntC: entorhinal cortex
EPSC: excitatory postsynaptic current
flox: floxed allele
FSI: fast-spiking interneuron
GLU: glutamate
Gls1: glutaminase 1
Hippo: hippocampus
IF: interfascicular nucleus
iGluR: ionotropic glutamate receptor
IHC: immunohistochemistry
IRES: internal ribosome entry site
ISH: *in situ* hybridization
mGluR: metabotropic glutamate receptor
MPP^+^: 1-methyl-4-phenyl pyridinium
MPTP: *N*-methyl -4-phenyl-1,2,3,6-tetrahydropyridine
m-shell: medial shell
nAChR: nicotinic acetylcholine receptor
NAc: nucleus accumbens
OT: olfactory tubercle
P#: postnatal day
PD: Parkinson disease
PBP: parabrachial pigmented nucleus
PFC: prefrontal cortex
PIF: parainterfascicular nucleus
PN: paranigral nucleus
RLi: rostral linear nucleus
sc RT-PCR: single cell reverse transcriptase polymerase chain reaction
SPN: spiny projection neuron
Shh: sonic hedgehog
SN: substantia nigra
SNc: substantia nigra pars compacta
SV: synaptic vesicle
TH: tyrosine hydroxylase
TH^+^/VGLUT^+^: TH and VGLUT2 double-labeling
US: unconditioned stimulus
VTA: ventral tegmental area
*VGluT2*: vesicular glutamate transporter 2 (rodent gene)
*VGLUT2*: vesicular glutamate transporter 2 (human gene)
VGLUT2: vesicular glutamate transporter 2 (protein)
VMAT2: vesicular monoamine transporter 2
WT: wildtype.
